# Relation of Flatfoot Severity with Flexibility and Isometric Strength of the Foot and Trunk Extensors in Children

**DOI:** 10.3390/children10010019

**Published:** 2022-12-22

**Authors:** Min Hwan Kim, Sangha Cha, Jae Eun Choi, Minsoo Jeon, Ja Young Choi, Shin-Seung Yang

**Affiliations:** Department of Rehabilitation Medicine, College of Medicine, Chungnam National University, Daejeon 35015, Republic of Korea

**Keywords:** pes planus, joint flexibility, pediatric, trunk strength, ankle strength

## Abstract

Background: Flatfoot is a deformity in which the foot is flattened due to a decrease in or loss of the medial longitudinal arch. Statement of the problem: Few studies have investigated the relationship between the severity of flat feet, trunk strength, and joint flexibility. Purpose: The aim of this study is to investigate the relationship between the severity of flatfoot and joint flexibility and foot and trunk strength in children with flexible flatfoot. Methods: This study included 16 children (boys, 12; girls, 4; age, 4~8 years) with flexible flatfeet. We examined the resting calcaneal stance position angle (RCSPA) and foot posture index (FPI) scores for clinical severity and radiographic parameters, such as calcaneal pitch angle, talometatarsal angle (TMA), and talocalcaneal angle (TCA). Muscle thicknesses of the tibialis posterior (TP), peroneus longus (PL), and L1 multifidus were measured by sonography. Isometric contraction of ankle inversion, eversion in a seating position, and lumbar extension at a prone position were induced using a handheld dynamometer to measure the maximum muscle strength for each muscle. Beighton’s scoring system was used to assess joint flexibility by evaluating the hyperextension of the joint for each category when performing stretching motion. Spearman’s rank correlation coefficient for nonparametric data was used. Results: The FPI showed a moderately negative correlation with the muscle thickness of TP (r = −0.558, *p* = 0.009) and L1 multifidus (r = −0.527, *p* = 0.012), and the strength of the ankle inverter (r = −0.580 *p* = 0.005) and lumbar extensor (r = −0.436 *p* = 0.043). RCSPA showed a moderately positive correlation with TCA (r = 0.510, *p* = 0.006). Beighton’s score showed no significant correlation with all parameters. Conclusion: In children with flatfoot, FPI reflected the clinical severity; thus, the more severe the symptoms, the weaker the ankle inverter and lumbar extensor.

## 1. Introduction

Flatfoot is a deformity which the foot is flattened due to a decrease in or loss of the medial longitudinal arch and is often accompanied by valgus of the hindfoot and supination of the forefoot [1]. Clinical symptoms of flexible flatfeet in children tend to improve as they grow up. However, in some children, various symptoms of flat foot such as pain, reduced exercise endurance, and abnormal gait may occur [2]. According to the previous study, the prevalence of flatfoot was reported to be 44% for children aged 3–6 years, 13.4 to 27.6% for elementary school students, and 13.6% for adolescents aged 18–21 years [3,4,5].

A flatfoot could be categorized into a rigid flatfoot and a flexible flatfoot. One study reported that the prevalence of flexible flatfoot was inversely proportional to the age, and it was more common in boys than in girls, and its incidence increased with weight gain [6]. Chen’s study reported that the joint laxity, W-sitting posture, the male sex, obese individuals, and young children had a higher risk of developing flat feet in preschool children aged 3–6 years [7,8].

The occurrence of flatfoot is due to several forces which flatten the arch and the little supporting structures for the maintaining the arch. The intrinsic muscle weakness and the laxity of the spring ligament, plantar fascia, or other supporting plantar ligaments were suggested as one of the mechanisms of the flexible flat foot [9].

Ligamentous laxity and joint hypermobility should be observed systemically in children with flexible flat foot. Generalized joint hypermobility could be related to the decreased muscle strength and motor performance. Children with generalized hypermobility often complained of being clumsy in early childhood and having difficulties in any participation in sporting and physical activities and they had the tendency to have severe flat foot. Especially, the reduced trunk muscle strength could negatively affect the spinal or lower body alignment and the physical performance in the hypermobile children with flat foot [6,10]. A few studies had been conducted on the relationship between the occurrence of flatfoot and the strength of the ankle. However, few studies have investigated the relationship between the severity of flat feet, trunk and ankle strength, and joint flexibility. Based on the above-mention, we hypothesized that the more severe the flatfoot, the lower the muscle strength of the foot and trunk and the higher the joint flexibility. Therefore, this study aimed to find the relationship between the severity of flatfoot and joint flexibility, as well as ankle and trunk strength in children with flexible flatfoot.

## 2. Materials and Methods

### 2.1. Participants

A total of patients (12 males and 4 females) who met the inclusion criteria who visited the hospital from April 2021 to June 2022 were included. The inclusion criteria were as follows: (1) a typical developing child aged 4−8 years; (2) diagnosed with flexible flatfoot having a resting calcaneal stance position angle (RCSPA) below −4°; (3) calcaneal pitch (CP) less than 18° [11]. The anthropometric data were collected at first and the maturation assessments were executed via the equation by Moore’s study [12]. We included participants in this study when they had only negative maturity offset and did not reach their peak height velocity. Patients with neurological abnormalities, anatomical abnormalities of the ankle and foot, genetic syndrome, or other neurodevelopmental disorders were also excluded from this study.

This study procedures were developed by the principles of the Declaration of Helsinki and were approved ethically by the Institutional Review Board of the Chungnam National University Hospital (No. 2019-12-001). The approval date of the ethic information was 28 January 2020. The researcher explained the overall contents of the study to the guardians and the child and obtained informed consent from them, which was approved by the institutional ethics committee.

### 2.2. Physical Examination

**Figure 1:**
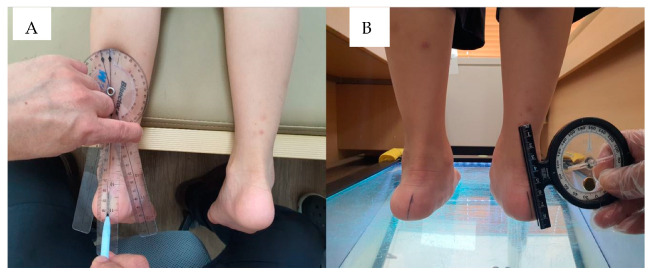
Measurement of the Resting Calcaneal Stance Position Angle (RCSPA) (**A**). Producing the centerline of the calcaneus (**B**). Measurement of RCSPA at a relaxed standing position.

RCSPA was measured to identify the clinical severity of flatfoot based on the method described by Root [13]. Patients were initially prone positioned on a bed parallel to the floor. The top, middle, and lower bisecting points of the calcaneus were marked, and the three locations were joined to produce a center line (Figure 1A). Patients maintained a relaxed stance with their feet separated by a distance equal to the width of an adult’s fist. The angle between the vertical line to the ground and the calcaneus centerline was calculated by a physician with more than 5 years of experience. (Figure 1B) A positive RCSPA indicates an inverted calcaneus, and a negative value suggests an everted calcaneus [11,14,15].

**Figure 2:**
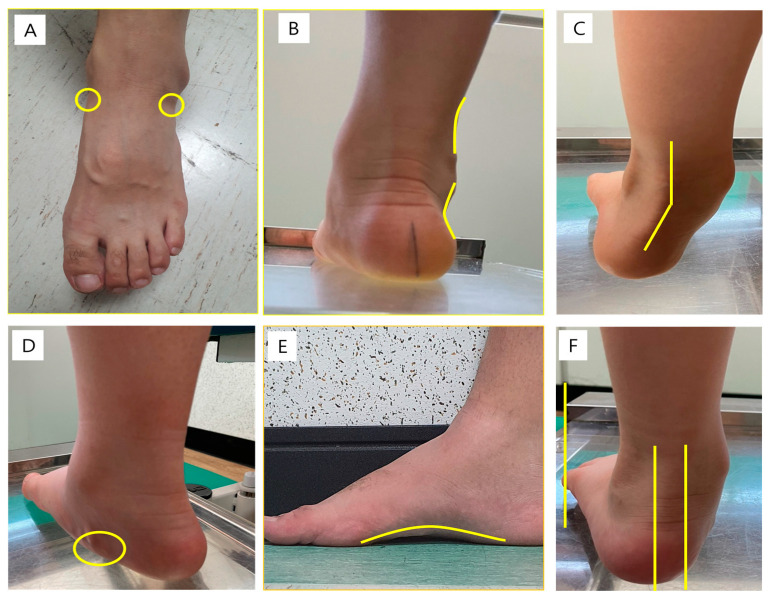
Measurements of the foot posture index (FPI). (**A**) Talar head palpation; (**B**) Supra- and infra-lateral malleolar curvature; (**C**) Calcaneal inversion/eversion; (**D**) Prominence of the talonavicular joint; (**E**) Congruence of the internal longitudinal arch; and (**F**) Abduction/Adduction of the forefoot with respect to the rear foot.

The foot posture index (FPI) was used to observe the shape of the foot, with scores from −2 to +2 according to the criteria for six relevant categories (Figure 2) (Table 1) [16]: (1) talar head palpation, (2) supra- and infra-lateral malleolar curvature, (3) calcaneus inversion/eversion, (4) prominence of the talonavicular joint, (5) congruence of the internal longitudinal arch, (6) abduction/adduction of the forefoot to the rear-foot. To determine the severity of the flatfoot, scores were summed and evaluated in a total ranging from −12 (highly supinated) to +12 (highly pronated) [17].

### 2.3. Radiographic Assessment of the Foot

**Figure 3:**
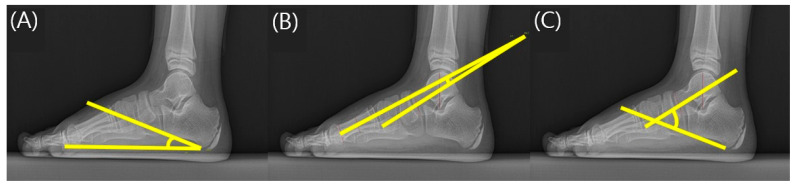
Measurement of radiographic parameters. (**A**) Calcaneal pitch angle; (**B**) Talometatarsal angle; (**C**) Talocalcaneal angle.

Foot radiographs were taken to evaluate the structural and anatomical deformities of the feet in flatfoot patients. To image the flatfoot, patients were asked to take off shoes and maintain a standing position in a weight-bearing position, and X-ray imaging of the foot was performed in the anteroposterior (AP) and lateral view. A previous study by Harris reported that there was a clinical correlation between calcaneal pitch angle (CPA), talometatarsal angle (TMA), and talocalcaneal angle (TCA) in children with flexible flatfoot [18]. The rehabilitation doctor with more than 3 years of experience measured the CPA, TMA, and TCA for each foot in the lateral view using PACS computer software according to the method mentioned by Sinha et al. [19] (Figure 3). The CPA measured the angle of the foot’s bottom line extending from the lower part of the calcaneocuboid joint to the lower boundary of the calcaneus and the line extending from the lower side of the medial calcaneus to the inferior boundary in the lateral view of the foot radiograph. The TMA was measured as the angle formed by the line that bisects the line tangent to the upper and lower edges of the talus at right angles and the line extending from the center of the metatarsal head. The TCA was measured as the angle between the line connecting the bottom boundary of the calcaneus and the line connecting the two center lines of the talus.

### 2.4. Thickness Assessment of the Foot and Trunk Muscle Using Ultrasonography

A trained physician with more than 5 years of experience performed the ultrasound scans using an ACUSON S2000 ultrasound unit (Siemens, Mount View, CA, USA) with a linear probe with a frequency of 9–14 MHz. To prevent the deformation of the muscle due to external pressure, the examiner applied a large amount of gel to the probe and measured the muscle with as little force as possible.

**Figure 4:**
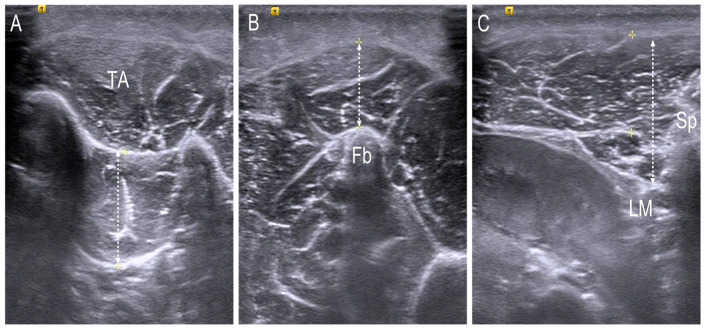
Anterior–posterior measurement of (**A**) tibialis posterior muscle; (**B**) peroneus longus muscle and (**C**) L1 Multifidus in the transverse scan. TA; tibialis anterior, Fb; fibula, LM; lamina, Sp; spinous process.

For obtaining the image of the tibialis posterior (TP) and peroneus longus (PL), the participants were asked to sit on the edge of a bed with flexed hip and knee positions. The images of the TP muscle as the inverter of the foot and the PL muscle as the evertor of the foot were obtained in a transverse view, and the length of each muscle was measured by using a measuring tool set on the ultrasound device. The scanning sites were based on the previous study [20] and were as follows: for the TP, one-third point of the imaginary line connecting from the medial malleolus and tibial tuberosity; for the PL, one-half point of the imaginary line connecting from the lateral malleolus to tibial tuberosity. Anterior–posterior measurements from the aponeurosis to the deepest fascia of the TP and from the superficial fascia of the PL to the bony surface of the fibula were executed, respectively. To obtain the image of the L1 multifidus, patients were asked to lay on their stomach with the forehead resting just above the breathing hole in the plinth, the head in the midline, and the arms outstretched. One pillow was placed under the hips to eliminate the lumbar lordosis [21]. The scan point was over the L1 multifidus next to the landmark of the L1 spinous process in a transverse plane and the distance between the muscle fascia below subcutaneous tissue and lamina was measured with the linear measuring tool (Figure 4). The muscle thickness was measured three times, and the mean value was used.

### 2.5. Isometric Strength Assessment of the Ankle and Trunk Muscle

The maximal isometric muscle strength of TP, PL, and L1 multifidus was measured with a portable hand-held digital dynamometer with integrated calibration (Power Track II Commander Muscle Tester: JTECH medical, Midvale, UT, USA). This method has been widely used in children and adolescents, and standardization studies for major muscles have been conducted in several countries. It has good intra-rater and inter-rater reliability for the assessment of isometric strength [22,23,24].

Before the assessment, the participants were asked to practice the corresponding movement first. The strength was measured via the breaking technique, in which the examiner commanded to push the device gradually without moving the assessed limb, and the maximal value was obtained when the child could maintain the constant force for more than 3 s. Tests were performed 3 times, respectively; 10 s of rest was allowed between each measurement. The average of the values was taken as the final value for each muscle group.

**Figure 5:**
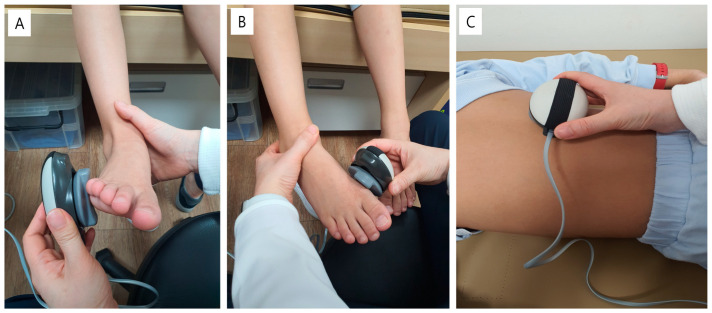
Measurement of isometric strength of ankle evertor (**A**), ankle inverter (**B**), and lumbar extensor (**C**) with a hand-held dynamometer.

For evaluating the strength of the ankle muscles, the participants were asked to sit on the edge of the bed with their hip and knee flexed at 90 degrees while the examiner held the ankle at the neutral position with one hand to prevent any compensating movement. The device was attached to the prominence of the first metatarsal head for measuring the strength of the ankle inverter (TP), and to the prominence of the fifth metatarsal head for measuring the strength of the ankle evertor (PL). To measure the strength of the lumbar extensor, the participants took a prone position in a relaxed state on the bed, and the device was attached to the spinous process of the L1 spine (Figure 5).

### 2.6. Flexibility of Joints

Beighton’s scoring system was used to determine the flexibility of the children. Participants were asked to bend their little finger back beyond 90 degrees, to bend their thumbs back to touch their forearms, to straighten their elbow past a neutral position, to straighten their knee past a neutral position, and to bend forward and place their hands flat on the floor without bending their knee: (1) pull the little finger back beyond 90°; (2) pull thumb back to touch the forearm; (3) bend the elbow backward beyond 10°; (4) bend the knee backward beyond 10°; (5) lie hands on the floor while keeping the knees straight and bending forward at the wrist [25]. These 5 specific functions were assessed for both little fingers, both thumbs, both elbows, both knees, and the trunk, and scored 1 for yes or 0 for no. If the score was more than 4 points out of the maximum 9, joint hypermobility was diagnosed.

Measurement of X-ray angle, ultrasound, isometric muscle strength, and flexibility was performed by a physician blinded to the condition of the children’s feet.

### 2.7. Statistical Analysis

Spearman’s rank correlation coefficient for nonparametric data was used to estimate the association of clinical parameters such as FPI and RCSPA, radiographic parameters, and trunk and ankle strength parameters using the Statistical Package for the Social Sciences for Windows (SPSS version 25.0, IBM SPSS Incorporated, Chicago, IL, USA). Spearman’s correlation of ≥0.80 was defined as very strong, 0.80 to 0.60 as strong, 0.60 to 0.40 as moderate, 0.40 to 0.20 as weak, and <0.20 as very weak. Statistical significance was set at *p* < 0.05. We also performed a correlation study according to the gender.

## 3. Results

Sixteen children (12 males and 4 females) with flexible flatfoot were recruited in this study. Table 2 shows the demographic characteristics of the recruited patients according to gender. The median age of total participants was 6 (minimum 4 and maximum 8) years old. The median age of males was 6 (minimum 4 and maximum 8) years old, and the median age of females was 5.5 (minimum 4 and maximum 6) years old. Two participants were up to 4 years old, 9 participants were in the range of 5–6 years old, and 5 participants were in the range of 7–8 years old.

Table 3 showed the clinical severities such as RCSPA and FPI score, and the values of radiographic and ultrasonographic measurements according to gender. Values are presented as mean ± standard deviation.

Table 4 showed the correlations between the muscle strength measured using a handheld dynamometer and the muscle thickness of the TP, PL, and L1 multifidus measured using ultrasounds for all participants. There was a strong positive correlation between the muscle thickness of TP and ankle inverter strength (r = 0.679, *p* = 0.000). Additionally, there was a moderate positive correlation between the muscle thickness of the L1 multifidus and lumbar extensor strength (r = 0.431, *p* = 0.014). In addition, Table 5 indicated the correlation between the muscle strength and the muscle thickness of the TP, PL, and L1 multifidus in the male participants. There was a strong positive correlation between the muscle thickness of the TP and ankle inverter strength (r = 0.653, *p* = 0.001), and a moderate positive correlation between the muscle thickness of the L1 multifidus and lumbar extensor strength (r = 0.413, *p* = 0.045). In this study, we inferred that muscle thickness measured by ultrasonography was proportional to muscle strength measured by the handheld dynamometer.

Table 6 showed the correlations between the clinical parameters such as FPI and RCSPA and the muscle thickness, muscle power, and radiologic parameters for all participants. In addition, Table 7 indicated the same correlations between the clinical parameters and the same objective measurements for male participants.

In the analysis of all participants, there was a moderate negative correlation between the muscle power of the ankle inverter (r = −0.580, *p* = 0.005) and the muscle thickness of the TP (r = −0.558, *p* = 0.009), and the FPI. There was also a moderate negative correlation between the FPI and the muscle thickness of the L1 multifidus (r = −0.527, *p* = 0.012) and the muscle power of the lumbar extensor (r = −0.436, *p* = 0.043) in all participants. There was also a moderate positive correlation between the RCSPA and TCA (r = 0.489, *p* = 0.005).

There was a moderate negative correlation between the FPI and the muscle thickness of the TP (r = −0.493, *p* = 0.044) and L1 multifidus (r = −0.507, *p*= 0.032), and there was a strong negative correlation between the muscle thickness of the ankle inverter (r = −0.603, *p* = 0.008), lumbar extensor (r = −0.632, *p* = 0.005), and FPI in male participants. There was also a moderate positive correlation between the RCSPA and TCA (r = 0.544, *p* = 0.006) in male participants.

Table 8 showed the correlation between the Beighton score, RCSP, FPI, radiologic parameters, muscle thickness, and muscle power in all participants, and Table 9 indicated the same analysis in male participants. There were no significant correlations between the Beighton score and any index in all participants and male participants.

## 4. Discussion

Many studies have been conducted on the diagnosis and treatment of flatfoot in children. This was the first study to investigate their association with the clinical severity of the flatfoot, ankle and trunk strength, muscle thickness, and the flexibility of joints in children with flexible flatfoot without neurological abnormality.

First, the FPI meaning the clinical severity of flatfoot, was inversely proportional to the power and the thickness of the TP muscle. The TP muscle is in the deep posterior compartment of the lower leg and inserted onto the navicular bone and the plantar slip is attached to the medial cuneiform bone. Thus, the TP muscle acts as the inverter and the plantar flexor of the ankle and supports the medial plantar arch. In several previous studies, the authors reported that the flexible flatfoot might develop when the weak TP could not maintain the medial longitudinal arch stable [26,27,28,29]. Therefore, this finding suggests that the strengthening exercise of the TP muscle can be one of managements for pediatric flexible flatfoot. However, in a previous study conducted by Shin et al. [14], they reported that the cross-sectional areas of the tibialis anterior and TP of the patients with flat feet were larger than these of the normal population. They explained that the ankle inverters were used to maintain the alignment of the foot arch against the tendency of the everted foot from the weight and ground reaction forces during the stance phase of gait, and such movement of the ankle might cause this muscular hypertrophy. The reason for this conflicting finding could be explained by the different ages of the enrolled subjects. Shin et al.’s study was focused on children over 12 years old on average, whereas our study included children who had not reached skeletal maturation with an average age of 6 years. In childhood, biological immaturities could lead to functional postural deviations such as flatfoot or kyphosis. Therefore, through this study, it can be inferred that strengthening of the lumbar extensor is necessary to prevent postural deviation in these school-aged children with flexible flatfoot.

Another interesting finding was that the power and the muscle thickness of the L1 multifidus were inversely correlated with the severity of flat feet such as the FPI. The TP and L1 multifidus muscle thickness was positively correlated with the corresponding muscle power. We could infer that the thickness of the muscle obtained from ultrasonography could reflect the muscle strength.

In a lower extremity closed kinetic chain, foot pronation is associated with internal rotation of the tibia and the femur, resulting in increased pelvic anterior displacement and lumbar lordosis [30,31]. Barwick et al. suggested that injuries related to pronated feet were associated with trunk and hip muscle weakness. They explained that the pathology related to excessive foot pronation was due to the weakness and dysfunction of the lumbopelvic–hip complex muscles [32]. In an adult study, the isokinetic concentric strength of hip flexors, extensors, internal rotators, and external rotators was decreased in patients with flatfoot, but the correlation between the concentric strength of trunk flexors and extensors and the severity of flatfoot has not been proven [33]. To the best of our knowledge, no previous study has compared the severity of flexible flatfoot with the muscle thickness of the lumbar spinal muscle in children. The results of our study showed that the flatter the foot, the smaller the size and strength of the lumbar extensors.

Clinically, we have found that children with flat feet tend to be more flexible. Therefore, this study was executed on the assumption that the children with the severity of the flatfoot would be correlated with the joint laxity. Although some previous studies had shown that there were positive correlations between joint laxity and the incidence of flatfoot in children, our result did not show the correlation between the degree of flexible flatfoot and the degree of joint flexibility as expressed by the Beighton score [34]. A previous study conducted on the correlation between joint hypermobility and flexible flatfoot in preschool-age flexible children concluded that there was no correlation between the Beighton score and the degree of flexible flatfoot, which was consistent with the results of our study [35]. They explained the reason for the negative result as follows: because the five specific postures of assessing the Beighton score were calculated by dichotomous answers (yes or no scores), each maneuver was not graded along a severity scale. Additionally, the temperature or other climate conditions could influence the results of assessing the flexibility. In our study, the average Beighton score was 8.25 ± 1.30, indicating that most children had a hypermobile joint so there would be a ceiling effect in the correlation study between the Beighton score and the severity of the flatfoot.

The TCA angle among the radiologic parameters was correlated with the severity of flatfoot expressed with the RCSPA, and other radiologic parameters were not correlated with the clinical parameters. A previous study reported that the RCSPA was correlated with the TMA [36]. The reason for the different findings might seem to arise from the difference in the enrolled subjects.

The first limitation of this study was the relatively small sample size. The number of subjects who visited the tertiary hospital with flexible flatfoot declined due to the outbreak of the COVID-19 pandemic, and the number of registered participants declined which caused bias in the data value.

The second limitation of this study was that it was conducted as an observational study, so cause and effect could not be identified.

Finally, the hand-held dynamometer could measure isometric muscular strength in increments of 2.2 newtons. Because of this technical limitation, there might have been a limit in accurately reflecting the children’s muscular strength and some clinical parameters might not be significantly correlated with muscle power.

In the future, it is necessary to recruit a larger number and wide age-range of children with flexible flatfeet. In addition, it is necessary to find out the change in the severity of flatfeet, joint flexibility, and muscular strength over the chronological change and skeletal maturation for a large number of children, and to analyze the correlation between these parameters.

The present study suggests that it is necessary to evaluate the muscular strength of the ankle inverter and lumbar extensor in children with flatfoot and to educate them about the importance of strengthening. Additionally, it is suggested that exercise to strengthen the TP and L1 multifidus might be one of the management strategies for children with flexible flatfeet.

## 5. Conclusions

The clinical severity of flatfoot measured with the FPI was correlated with a weak ankle inverter muscle and a weak lumbar extensor. Our results suggest that strengthening exercise for the ankle inverter and lumbar extensor might be needed in school-aged children with flexible flat feet.

## Figures and Tables

**Table 1:** Foot Posture Index scoring.

	−2	−1	0	1	2
Talar head palpation	Talar head palpable on the lateral side/but not on the medial side	Talar head palpable on the lateral side/ slightly palpable on the medial side	Talar head equally palpable on the lateral and medial side	Talar head slightly palpable on the lateral side/palpable on the medial side	Talar head not palpable on the lateral side/but palpable on the medial side
Supra- and infra-lateral malleolar curvature	Curve below the malleolus either straight or convex	Curve below the malleolus concave, but flatter/more than the curve above the malleolus	Both infra- and supra-malleolar curves roughly equal	Curve below the malleolus more concave than the curve above the malleolus	Curve below the malleolus markedly more concave than the curve above the malleolus
Calcaneal frontal plane position	More than an estimated 5° inverted (varus)	Between vertical and an estimated 5° inverted (varus)	Vertical	Between vertical and an estimated 5° everted (valgus)	More than an estimated 5° everted (valgus)
Prominence in the region of the talonavicular joint (TNJ)	Area of the TNJ markedly concave	Area of the TNJ slightly, but definitely, concave	Area of the TNJ flat	Area of the TNJ bulging slightly	Area of the TNJ bulging markedly
Congruence of the medial longitudinal arch	Arch high and acutely angled towards the posterior end of the medial arch	Arch moderately high and slightly acute posteriorly	Arch height normal and concentrically curved	Arch lowered with some flattening in the central position	Arch very low with severe flattening in the central position—arch making ground contact
Abduction/adduction of the forefoot on the rare foot	No lateral toes visible; medial toes clearly visible	Medial toes clearly more visible than the lateral	Medial and lateral toes equally visible	Lateral toes clearly more visible than medial	No medial toes visible; lateral toes clearly visible

**Table 2:** Demographic characteristics of participants according to the gender.

Characteristics		Total (n = 16)	Male (n = 12)	Female (n = 4)
Age (year)	Median Interquartile range Minimum Maximum	6 5–7 4 8	6 5–7 4 8	5.5 4.75–6 4 6
Height (cm)	Median Interquartile range Minimum Maximum	119.4 106.1–123.8 100.3 135.5	119.4 106.1–123.8 100.3 135.5	120.35 115.25–122.45 104 124.7
Body weight (kg)	Median Interquartile range Minimum Maximum	21.6 16.8–25.9 15.8 43.4	21 16.5–23.3 15.8 43.4	25.75 23.3–26.5 16.7 28
BMI	Median Interquartile range Minimum Maximum	15.46 14.9–17.2 14 23.63	15.01 14.4–16.0 14 23.63	17.78 17–18 16.7 18
Maturity offset	Mean ± SD	−5.28 ± 0.57	−5.53 ± 0.61	−5.04 ± 0.56

BMI, body mass index; Female maturity offset = −7.709133 + (0.0042232 × (age×height)); Male maturity offset = −7.999994 + (0.0036124 × (age×stature)).

**Table 3:** Clinical parameters and values of radiologic and ultrasonographic measurements according to the gender.

Characteristics	Total (n = 16)	Male (n = 12) Mean ± SD	Female (n = 4)
RCSPA (°)	−7.94 ± 2.37	−8.08 ± 2.22	−7.5 ± 2.74
FPI score (points)	9.45 ± 1.44	9.78 ± 1.31	8.0 ± 1.0
Beighton’s score (points)	8.43 ± 0.98	8.5 ± 0.81	8.25 ± 1.30
Muscle thickness (mm)			
Tibialis posterior	17.08 ± 3.07	17.20 ± 3.35	16.73 ± 2.05
Peroneus longus	11.32 ± 2.74	11.33 ± 2.92	11.30 ± 2.11
L1 multifidus	15.68 ± 2.25	16.04 ± 2.22	14.61 ± 2.01
Muscle power (N)			
Ankle inverter	12.08 ± 4.62	12.01 ± 4.89	12.28 ± 3.64
Ankle evertor	10.52 ± 3.20	10.82 ± 3.35	9.63 ± 2.51
L1 multifidus	10.90 ± 2.70	11.24 ± 2.88	9.90 ± 1.68
Radiographic parameter (°)			
CPA	11.00 ± 2.37	11.13 ± 2.53	10.64 ± 1.73
TMA	22.81 ± 5.31	22.37 ± 5.28	24.15 ± 5.15
TCA	51.74 ± 4.09	51.38 ± 3.84	52.83 ± 4.60

**Table 4:** Correlation between muscle strength and muscle thickness for all participants (n = 16).

Muscle Thickness/Muscle Power	Correlation	*p*-Value
Tibialis posterior/ankle inverter	0.679 **	0.000
Peroneus longus/ankle evertor	0.151	0.418
L1 multifidus/lumbar	0.431 *	0.014

* Correlation is significant at the 0.05 level by the Spearman correlation coefficient; ** Correlation is significant at the 0.01 level by the Spearman correlation coefficient.

**Table 5:** Correlation between muscle strength and muscle thickness for male participants (n = 12).

Muscle Thickness/Muscle Power	Correlation	*p*-Value
Tibialis posterior/ankle inverter	0.653 **	0.001
Peroneus longus/ankle evertor	0.080	0.718
L1 multifidus/lumbar	0.413 *	0.045

* Correlation is significant at the 0.05 level by the Spearman correlation coefficient; ** Correlation is significant at the 0.01 level by the Spearman correlation coefficient.

**Table 6:** Correlation between clinical parameters and muscle thickness, muscle power, and the radiologic parameters for all participants (n = 16).

	FPI		RCSPA	
	Correlation	*p*-Value	Correlation	*p*-Value
Muscle Thickness				
TP	−0.558 **	0.009	0.258	0.162
PL	−0.220	0.338	−0.118	0.526
L1 multifidus	−0.527 *	0.012	−0.177	0.331
Muscle power				
Ankle inverter	−0.580 **	0.005	0.010	0.956
Ankle evertor	−0.257	0.249	−0.044	0.809
Lumbar	−0.436 *	0.043	0.245	0.176
Radiologic parameter				
CPA	−0.290	0.191	0.165	0.368
TMA	−0.214	0.339	0.017	0.925
TCA	−0.078	0.730	0.489 **	0.005

* Correlation is significant at the 0.05 level by the Spearman correlation coefficient; ** Correlation is significant at the 0.01 level by the Spearman correlation coefficient.

**Table 7:** Correlation between clinical parameters and muscle thickness, muscle power, and the radiologic parameters for male participants (n = 12).

	FPI		RCSPA	
	Correlation	*p*-Value	Correlation	*p*-Value
Muscle Thickness				
TP	−0.493 *	0.044	0.375	0.078
PL	−0.127	0.628	0.018	0.935
L1 multifidus	−0.507 *	0.032	−0.020	0.927
Muscle power				
Ankle inverter	−0.603 **	0.008	0.124	0.565
Ankle everter	−0.447	0.063	0.043	0.841
Lumbar	−0.632 **	0.005	0.330	0.115
Radiologic parameter				
CPA	−0.372	0.129	0.296	0.160
TMA	−0.163	0.517	−0.044	0.838
TCA	−0.098	0.699	0.544 **	0.006

FPI, foot posture index; RCSPA, resting calcaneal stance position angle; TP, Tibialis posterior, PL, Peroneus longus; TMA, Talometatarsal angle; TCA, Talocalcaneal angle; CPA, Calcaneal pitch angle. * Correlation is significant at the 0.05 level by the Spearman correlation coefficient; ** Correlation is significant at the 0.01 level by the Spearman correlation coefficient.

**Table 8:** Correlation between Beighton score and RCSP, FPI, radiologic parameters, muscle thickness, and muscle power of flatfoot for all participants (n = 16).

	Beighton Score (Flexibility)	
	Correlation	*p*-Value
RCSPA	0.136	0.491
FPI	0.378	0.122
Radiologic parameter		
CPA	−0.023	0.907
TMA	0.308	0.110
TCA	0.054	0.783
Muscle thickness		
TP	−0.071	0.720
PL	−0.019	0.925
L1 multifidus	−0.372	0.051
Muscle power		
Ankle inverter	0.008	0.967
Ankle everter	0.242	0.215
Lumbar	0.077	0.698

**Table 9:** Correlation between Beighton score and RCSP, FPI, radiologic parameters, muscle thickness, and muscle power of flatfoot for male participants (n = 12).

	Beighton Score (Flexibility)	
	Correlation	*p*-Value
RCSPA	0.241	0.307
FPI	0.000	1.000
Radiologic parameter		
CPA	−0.252	0.284
TMA	0.422	0.063
TCA	0.025	0.916
Muscle thickness		
TP	0.203	0.390
PL	−0.226	0.339
L1 multifidus	−0.387	0.089
Muscle power		
Ankle inverter	0.227	0.335
Ankle evertor	0.411	0.072
Lumbar	0.129	0.587

## Data Availability

The data presented in this study are available on request from the corresponding author.
